# Automatic segmentation of cotton roots in high-resolution minirhizotron images based on improved OCRNet

**DOI:** 10.3389/fpls.2023.1147034

**Published:** 2023-05-10

**Authors:** Yuxian Huang, Jingkun Yan, Yuan Zhang, Weixin Ye, Chu Zhang, Pan Gao, Xin Lv

**Affiliations:** ^1^ College of Agriculture, Shihezi University, Shihezi, China; ^2^ College of Information Science and Technology, Shihezi University, Shihezi, China; ^3^ School of Information Engineering, Huzhou University, Huzhou, China

**Keywords:** plant root, image processing, computer vision, semantic segmentation, attention mechanism

## Abstract

Root phenotypic parameters are the important basis for studying the growth state of plants, and root researchers obtain root phenotypic parameters mainly by analyzing root images. With the development of image processing technology, automatic analysis of root phenotypic parameters has become possible. And the automatic segmentation of roots in images is the basis for the automatic analysis of root phenotypic parameters. We collected high-resolution images of cotton roots in a real soil environment using minirhizotrons. The background noise of the minirhizotron images is extremely complex and affects the accuracy of the automatic segmentation of the roots. In order to reduce the influence of the background noise, we improved OCRNet by adding a Global Attention Mechanism (GAM) module to OCRNet to enhance the focus of the model on the root targets. The improved OCRNet model in this paper achieved automatic segmentation of roots in the soil and performed well in the root segmentation of the high-resolution minirhizotron images, achieving an accuracy of 0.9866, a recall of 0.9419, a precision of 0.8887, an F1 score of 0.9146 and an Intersection over Union (IoU) of 0.8426. The method provided a new approach to automatic and accurate root segmentation of high-resolution minirhizotron images.

## 1 Introduction

The root system is the nutrient organ of plants, which plays an important role in promoting plant growth. In root studies, the root phenotypic parameter is an important measure of root growth status. Since soil is an opaque medium, it is not possible to survey roots directly to obtain root phenotypic parameters, except by some methods. Excavation is a traditional method of obtaining roots that can expose the roots to the soil for the purpose of direct root survey and can be divided into methods such as shovelomics method (Trachsel et al., 2011), core method (Wasson et al., 2016) and trench profile method (Faye et al., 2019). But excavation is a destructive sampling method and also requires considerable time and labor. Hydroponics can be selected to cultivate plants in order to monitor their growth status and obtain plant root information (Li et al., 2018). Although root information is more readily available through hydroponic cultivation of plants than through excavation, the findings from studies using hydroponics cannot be generalized to root studies in the soil environments. In order to obtain root information quickly and easily in the soil environments without damaging plants, root researchers have used sensors to monitor plant roots non-destructively, such as X-ray computed tomography (CT) to reconstruct the three-dimensional structure of rice roots (Teramoto et al., 2020), nuclear magnetic resonance (NMR) imaging technology to analyze the root structure of wheat (Pflugfelder et al., 2022), and minirhizotrons to photograph cotton roots (Shen et al., 2020). Although all of these methods can obtain *in situ* non-destructive images of roots, these methods are applicable to different scenarios. The implementation of CT and NMR imaging technology requires expensive and highly technical equipment, and both methods are in most cases only applicable to the observation of plant roots in small potted plants. In contrast, minirhizotrons are less expensive and simpler to operate, and minirhizotrons can be inserted into the soil in the field to observe the roots. Therefore, minirhizotrons are well suited to be used to collect *in situ* images of plant roots in the field to analyze root phenotypic parameters.

Traditional root segmentation is achieved manually by image processing software which is time-consuming and labor-intensive. To address the drawbacks of manual root segmentation, machine learning methods are applied by root researchers for automatic root segmentation. Machine learning is a method that allows machines to simulate or learn human behavior, and common machine learning methods include OTSU (Otsu, 1979), Support Vector Machine (SVM) (Cortes and Vapnik, 1995) and Random Forests (Breiman, 2001). A segmentation method based on color features of the roots of wheat seedlings was implemented: firstly, the root image was converted from RGB color space to HCI color space, then the threshold of the chroma component was set to extract the binary image, and finally, the image was processed with local fuzzy c-means clustering algorithm to get the segmentation result (Goclawski et al., 2009). The OTSU method was applied to the study of automatic root segmentation of the images acquired by desktop scanners, and this method is an image segmentation algorithm based on a dynamic threshold (Chen and Zhou, 2010). The threshold segmentation methods are usually only applicable to the automatic segmentation of root images with simple backgrounds. Moreover, the threshold segmentation methods need to be set with suitable thresholds in advance, which leads to the poor generalization of the threshold segmentation methods.

The Convolutional Neural Network (CNN) is a method proposed to compensate for the previous machine learning’s inability to learn autonomously like the human brain. LeNet-5 (Lecun et al., 1998), as one of the earliest CNNs, successfully realized the recognition of handwritten fonts. Later, the CNN-based AlexNet (Krizhevsky et al., 2012) was proposed to achieve the automatic classification of images. The proposal of FCN (Long et al., 2015) gave CNNs the ability of semantic segmentation. Then, excellent semantic segmentation methods such as U-Net (Ronneberge et al., 2015), PSPNet (Zhao et al., 2017), DeepLabv3+ (Chen et al., 2018) and OCRNet (Yuan et al., 2020) were created. With the development of image segmentation algorithms for deep learning, many root researchers have applied CNN-based image segmentation models in automatic root segmentation research. An improved DeepLabv3+ model was used for the automatic segmentation of cotton roots, where root images were acquired from minirhizotrons installed in the field, and experimental results showed that this improved semantic segmentation model worked well for root segmentation of minirhizotron images with a real soil environment as the background (Shen et al., 2020). U-Net was applied to the segmentation of soybean seedling roots, and experimental results showed that the method could achieve accurate segmentation of soybean seedling roots (Xu et al., 2022). CNN-based segmentation methods have been widely used in automatic root segmentation research and have achieved good performance. These methods not only do not need to be set with suitable thresholds in advance but also can be applied to root segmentation in real soil environments. To further improve the performance of semantic segmentation models, many root researchers have added attention mechanisms to the segmentation models to enhance the root segmentation capability of the models.

The attention mechanism is an information processing mechanism that focuses on useful information and ignores useless information. The classical attention mechanisms contain Squeeze-and-Excitation (SE) (Hu et al., 2018), Convolutional Block Attention Module (CBAM) (Woo et al., 2018) and Non-Local (Wang et al., 2018). The recently proposed attention mechanisms contain Efficient Channel Attention (ECA) (Wang et al., 2020), Coordinate Attention (CA) (Hou et al., 2021) and Global Attention Mechanism (GAM) (Liu et al., 2021). Since the attention mechanism can enhance the focus of the segmentation model on the root targets, the attention mechanism has been applied in some root segmentation studies for improving the segmentation model and enhancing the root segmentation capability of the model. For example, an improved U-Net with the SE attention module was applied to the study of rice root segmentation, which achieved automatic precision segmentation of rice seedling roots in the images (Gong et al., 2021).

Nowadays, most of the root segmentation researchers still use DeepLabv3+ and the previous semantic segmentation models. As a model proposed in recent years, OCRNet uses the object region to which each pixel belongs as the region for extracting contextual representation, which is a better way to obtain contextual representation for each pixel than DeepLabv3+. Moreover, the background of *in situ* images is a real soil environment, and there are many noises in the soil that interfere with automatic root segmentation, and these noises will affect the accuracy of automatic root segmentation. An attention mechanism can be added to the model, which can enhance the model’s attention to the root targets and improve the model’s ability to distinguish the roots from the background. Therefore, this work aims to explore the application of an advanced semantic segmentation network model improved by an attention mechanism for cotton root segmentation of minirhizotron images with a real soil environment as the background. The specific objectives achieved here are as follows:

(1) Collect high-resolution *in situ* cotton root images using minirhizotrons and annotate these images.
(2) Improve OCRNet by adding the GAM attention module to optimize the pixel representations output by the backbone.
(3) Compare and evaluate the improved method with the mainstream semantic segmentation methods.

## 2 Materials and methods

### 2.1 Data collection

The high-resolution minirhizotron images were collected at the experimental station of Shihezi University College of Agriculture in Shihezi, Xinjiang Uygur Autonomous Region (85°59′43.7064″E, 44°19′21.1044″N). In 2018, we selected two areas in the cotton field, installed two minirhizotrons in each area, and collected data eight times (July 6, July 10, July 14, July 18, July 22, July 26, July 30, and August 3). Each minirhizotron was used to scan cotton roots at three different depths at a time. We acquired a total of 96 high-resolution minirhizotron images, each with a size of 2271 × 2550 pixels.

### 2.2 Data annotation

**Figure 1:**
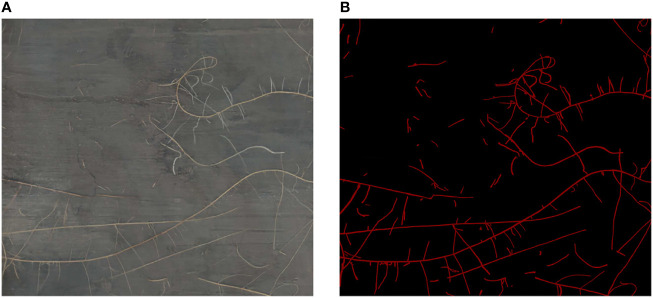
Data Annotation. **(A)** Original image. **(B)** Annotated image.

We screened the high-quality images among these 96 high-resolution minirhizotron images. In this process, we removed four images that were blurred, leaving 92 high-quality root images. We used the LabelMe 3.16.7 (Russell et al., 2008) annotation tool to annotate these 92 high-resolution minirhizotron images and generate corresponding annotated images, with the roots marked in red and the background in black (**Figure 1**). The average annotation time per image was 8 hours.

### 2.3 Data augmentation

In order to expand the dataset and improve the generalization ability of the model obtained from subsequent training, we performed data augmentation on these 92 high-resolution minirhizotron images after data annotation was completed. We used five ways of data augmentation, namely, increasing luminance, decreasing luminance, isometric enlargement, isometric reduction, and adding salt-and-pepper noise. Finally, the number of images was expanded to 552, and the number of annotated images was also expanded to 552 accordingly to form the final high-resolution minirhizotron image dataset. We generated 460 new annotated images from 92 annotated images by corresponding transformation operations according to the data augmentation, so the 460 new images generated from 92 original images by data augmentation do not need to be manually annotated again. We divided the 552 high-resolution minirhizotron images into the training set, the validation set and the test set in the ratio of 6: 2: 2. There are 330 images in the training set, 111 images in the validation set and 111 images in the test set. The training set was used for training the network model, and the validation set was used to select the model weights that performed best during model training to evaluate the model performance by using the test set.

### 2.4 Segmentation model

OCRNet is a semantic segmentation method that makes the classification of each pixel and the segmentation of each class more accurate by augmenting the representation of each pixel with the object-contextual representation (OCR) (Yuan et al., 2020). We applied the OCRNet semantic segmentation model to the root segmentation of high-resolution minirhizotron images. In the semantic segmentation model, the backbone plays the role of representation extraction which has an important impact on the performance of the segmentation model.

**Figure 2:**
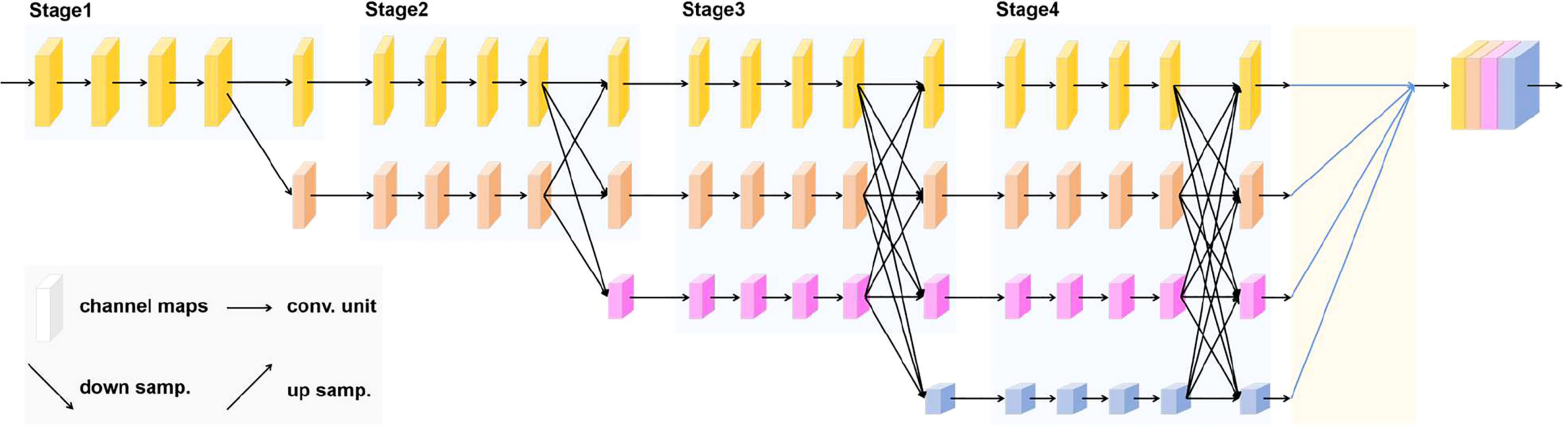
HRNetV2 network structure.

HRNetV2 (Sun et al., 2019a) is a network that retains high-resolution representations well and is well suited for segmentation of high-resolution images with elongated or tiny objects. In the high-resolution minirhizotron image dataset we acquired, the resolution of each image is relatively high, and the roots in each image are very fine, so the model used must be able to notice the minute details of the roots to achieve accurate segmentation of it. Therefore, HRNetV2 is well suited for extracting root representations from high-resolution minirhizotron images. We used HRNetV2 as the backbone of OCRNet to extract pixel representations. HRNetV2 contains four stages with four parallel subnetworks. In HRNetV2, the resolution is gradually decreased to a half and accordingly the number of channels is increased to the double. The first stage contains 4 residual units where each unit, the same to the ResNet-50 (He et al., 2016), is formed by a bottleneck with 64 channels, and is followed by one 3×3 convolution reducing the number of channels of feature maps to C (C represents the number of channels of the high-resolution subnetworks in the last three stages). The 2nd, 3rd and 4th stages contain 1, 4 and 3 exchange blocks, respectively. One exchange block contains 4 residual units where each unit contains two 3 × 3 convolutions in each resolution and an exchange unit across resolutions. In summary, there are a total of 8 exchange units, i.e., 8 multi-scale fusions are conducted. Unlike HRNetV1 (Sun et al., 2019b), HRNetV2 concatenates the representations of the four resolution subnetworks in the last stage, making full use of the representations of each resolution subnetwork. The network structure of HRNetV2 is shown in **Figure 2**. We chose HRNetV2-W48 as the final backbone, where 48 represents the number of channels (C) of the high-resolution subnetworks in the last three stages. The number of channels in the other three parallel subnetworks is 96, 192 and 384 for HRNetV2-W48.

After extracting the pixel representations by using HRNetV2-W48 as the backbone of OCRNet, a coarse semantic segmentation result is output by FCN to obtain K soft object regions (soft object region refers to the region consisting of pixels of each class in the coarse semantic segmentation result). K equals 2 because there are only two classes in the annotation of our high-resolution minirhizotron images, i.e., background and root. After obtaining the soft object regions, the pixel representations output from the backbone and the semantic representations of the corresponding K soft object regions are weighted and summed to obtain the K object region representations. The calculation of each object region representation is shown as follows:


(1) $$f_{k}=\sum_{i\in I}{\tilde{m}}_{ki}x_{i}$$

where ${\text{f}}_{\text{k}}$ is the representation of the k th object region. ${\text{x}}_{\text{i}}$ is the representation of pixel i . I refers to the set of pixels in the image. ${\tilde{\text{m}}}_{\text{ki}}$ is the normalized degree for pixel i belonging to the k th object region.

Then, the pixel-region relation is obtained by computing the relation between each pixel and each object region according to the self-attention (Vaswani et al., 2017), which is shown as follows:


(2) $$w_{ik}=\frac{e^{k\left(x_{i},\;f_{k}\right)}}{\sum_{j=1}^{K}e^{k\left(x_{i},\;f_{j}\right)}}$$

where ${\text{w}}_{\text{ik}}$ is the relation between the i th pixel and the k th object region. $\text{k}\left({\text{x}}_{\text{i}},\;{\text{f}}_{\text{i}}\right)=\emptyset {\left(x\right)}^{⊤}\text{ψ}\left(\text{f}\right)$ is the unnormalized relation function, ∅(·) and ψ(·) are two transformation functions consisting of a 1×1 convolution layer, a Batch Normalization (BN) layer and a ReLU function. K is the number of object regions.

After obtaining the pixel-region relation, the object contextual representations are calculated based on the pixel-region relation and the object region representations. The calculation of the object contextual representation for each pixel is shown as follows:


(3) $$y_{i}=\rho \left(\sum_{k=1}^{K}w_{ik}\delta \left(f_{k}\right)\right)$$

where ${\text{y}}_{\text{i}}$ is the object contextual representation of pixel i . ρ(·) and δ(·) are two transformation functions consisting of a 1×1 convolution layer, a BN layer and a ReLU function. K is the number of object regions. ${\text{w}}_{\text{ik}}$ is the relation between the i th pixel and the k th object region. ${\text{f}}_{\text{k}}$ is the representation of the k th object region.

**Figure 3:**
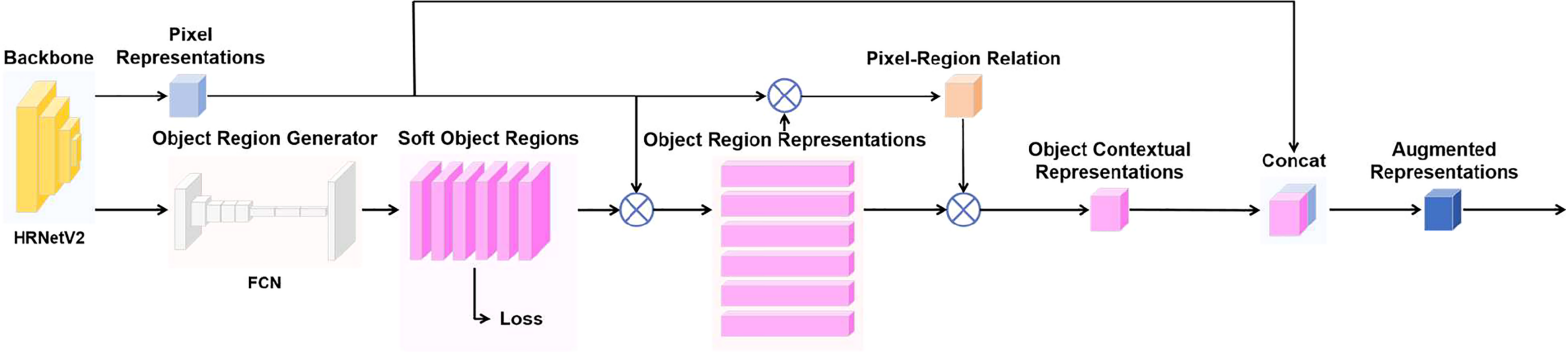
OCR module.

Finally, the augmented representation for each pixel is calculated as the aggregation of the original representation for each pixel and the object contextual representation for each pixel, which is shown as follows:


(4) $$z_{i}=g({\left[x_{i}^{\;⊤}y_{i}^{\;⊤}\right]}^{⊤})$$

where ${\text{z}}_{\text{i}}$ is the augmented representation for pixel i . g(·) is a transformation function used to fuse the original representation and the object contextual representation, consisting of a 1×1 convolution layer, a BN layer and a ReLU function. ${\text{x}}_{\text{i}}$ is the representation of pixel i . ${\text{y}}_{\text{i}}$ is the object contextual representation of pixel i . The whole OCR module takes the pixel representations output by the backbone as input and the augmented representations as output, as illustrated in **Figure 3**. After obtaining the augmented representations, the output with the number of classes equal to the number of channels is obtained by a 1×1 convolution layer, and then the output is restored to the original scale by bilinear up-sampling to obtain the final semantic segmentation prediction result.

### 2.5 Model improvement

GAM is a global attention mechanism that focuses on the interaction of the three dimensions of widths, heights, and the number of channels in the feature maps (Liu et al., 2021). Therefore, GAM reduces information reduction and magnifies global dimension-interactive features, which allows the network model to focus on the features of the targets in a comprehensive manner. In our collected high-resolution minirhizotron images, the color of the roots is similar to the color of the soil in the background, which causes the model to have more difficulty in distinguishing the roots from the soil in the background. And the images we collected include a variety of complex background noises, such as stones, worms, soil cracks, residual plastic film, etc. These background noises can interfere with the model in distinguishing the roots from the background. And enhancing the model’s focus on the roots is a key way to improve the model’s ability to distinguish the roots from the background. Because of the role of making the model to focus on the features of the targets in a comprehensive manner, the addition of the GAM module in the model can enhance the model’s focus on the roots to improve the model’s ability to distinguish the roots from the background. Therefore, we improved OCRNet by adding the GAM module. And by this improvement, the model’s ability to identify and segment the roots can be improved. GAM follows the structure of CBAM in which the channel attention submodule and spatial attention submodule are connected in series. But GAM redesigned the channel attention submodule and the spatial attention submodule in the structure. In GAM, given an input feature map, GAM outputs a three-dimensional channel attention map through the channel attention submodule, multiplies the input and output of the channel attention submodule to obtain the input of the spatial attention submodule, and then outputs a three-dimensional spatial attention map, and multiplies the input and output of the spatial attention submodule to obtain the final output. The overall attention process can be summarized as:


(5) $$F_{2}=M_{C}(F_{1})⊗F_{1}$$

(6) $$F_{3}=M_{S}(F_{2})⊗F_{2}$$

where ⊗ denotes element-wise multiplication. ${\text{F}}_{1}$ is the feature map input by GAM and ${\text{F}}_{1}\in {\mathbb{R}}^{\text{C}\times \text{H}\times \text{W}}$ . ${\text{M}}_{\text{C}}$ is the output of the channel attention submodule of GAM and ${\text{M}}_{\text{C}}\in {\mathbb{R}}^{\text{C}\times \text{H}\times \text{W}}$ . ${\text{F}}_{2}$ is the result of multiplying ${\text{M}}_{C}$ and ${\text{F}}_{1}$ . ${\text{M}}_{\text{S}}$ is the output of the spatial attention submodule of GAM and ${\text{M}}_{\text{S}}\in {\mathbb{R}}^{\text{C}\times \text{H}\times \text{W}}$ . ${\text{F}}_{3}$ is the final output of GAM.

**Figure 4:**
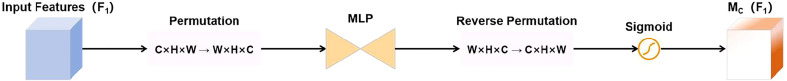
Channel attention submodule of GAM.

In the channel attention submodule of GAM, the 3D information of the feature map is retained by a 3D permutation module, magnified by a two-layer Multi-Layer Perceptron (MLP), converted into 3D information in the original dimensional order by a 3D reverse permutation module, and finally input into a sigmoid function to obtain the channel attention map. The structure of the channel attention submodule of GAM is shown in **Figure 4**.

**Figure 5:**
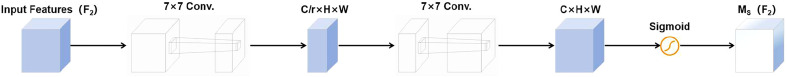
Spatial attention submodule of GAM.

In the spatial attention submodule of GAM, the number of channels of the feature map is first reduced to C/r by a 7×7 convolutional layer, followed by a 7×7 convolutional layer to restore the number of channels to C, and finally input into a sigmoid function to obtain the spatial attention map. The structure of the spatial attention submodule of GAM is shown in **Figure 5**.

**Figure 6:**
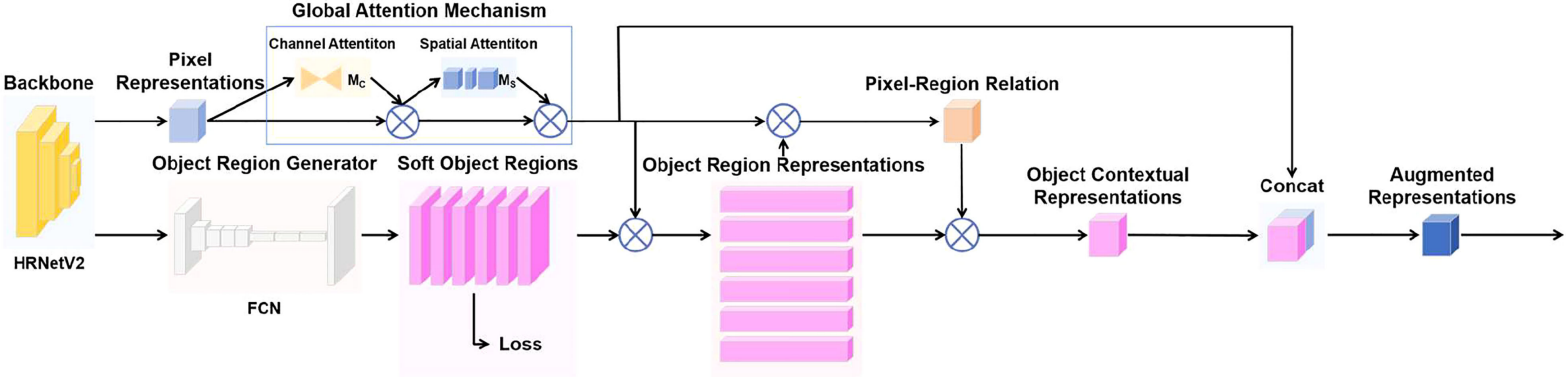
Improved OCR module.

The pixel representations are involved in the computation several times throughout the OCR module, affecting the individual outputs in the module (**Figure 5**). It shows that the pixel representations are closely related to the segmentation effect of OCRNet. In order to optimize the pixel representations and thus improve the segmentation effect of OCRNet, we improved the OCR module by adding the GAM module. In the improved OCR module, the pixel representations are input to the GAM module, and the GAM module outputs the optimized pixel representations. Instead of the original pixel representations, the optimized pixel representations participate in the computation of the object region representations, the pixel-region relation, and the object contextual representations. The improved OCR module is shown in **Figure 6**.

### 2.6 Network training

The OCRNet we designed has two outputs, one is the coarse semantic segmentation result output by using FCN as the object region generator, and the other is the final prediction result output by the whole model. For both outputs, we used two pixel-wise cross-entropy loss functions to calculate these two loss values separately, with the loss weight set to 0.4 for the former and 1 for the latter. Since the root segmentation is a pixel-level binary classification problem, the calculation of the cross-entropy loss is shown as follows:


(7) $$Loss=-\frac{1}{N}\sum_{i=1}^{N}\left[y_{i}lnp_{i}+\left(1-y_{i}\right)ln\left(1-p_{i}\right)\right]$$

where N is the number of pixels, ${\text{y}}_{\text{i}}$ is the label of pixel i , and ${\text{p}}_{\text{i}}$ is the predicted probability value.

Due to the limited GPU memory and the fact that the model parameters, gradients, optimizer states and intermedia activations cost the GPU memory during training, the size of the images input to the network model during training cannot be too large. During the training process, we set up a random crop pipeline so that the high-resolution original images were randomly cropped into sub-images of size 512 × 512 pixels before being input into the network model. We used the polynomial decay method to achieve the decay of the learning rate, the initial learning rate was set to 0.01, the power of the polynomial was set to 0.9, and the minimum learning rate was set to 0.0001. In order to make the model training more stable and converge better, we used the SGDM optimizer with momentum set to 0.9 and weight decay set to 0.0005. We set the batch size to 4 and the total number of iterations to 40,000. In the process of training the network model, we saved the model weights every 500 iterations, tested the model weights using the validation set, and selected the best-performing model weights to evaluate the model performance by using the test set. The detailed hyperparameters during network training are shown in **Table 1**.

**Table 1:** Hyperparameters during network training.

Hyperparameters	Value
Loss function	Cross-entropy loss
Random crop size	512 × 512 pixels
Input size	512 × 512 pixels
Learning rate decay strategy	Polynomial decay
Initial learning rate	0.01
Power of polynomial decay	0.9
Minimum learning rate	0.0001
Optimizer	SGDM
Momentum of SGDM	0.9
Weight decay	0.0005
Batch size	4
Max iterations	40000

The server environment was Windows 10, and the program was compiled and run in Python 3.7. The model was trained, validated, and tested under PyTorch 1.8.1 and CUDA 11.1. The server was equipped with an NVIDIA GeForce RTX 3080 Laptop (16G) graphics card for model training acceleration.

### 2.7 Evaluation

In order to objectively and reasonably evaluate the root segmentation performance of our model, we utilized five evaluation metrics, i.e., accuracy, recall, precision, F1 score, and Intersection over Union (IoU):


(8) $$Accuracy=\frac{TP+TN}{TP+FP+FN+TN}$$

(9) $$Recall=\frac{TP}{TP+FN}$$

(10) $$Precision=\frac{TP}{TP+FP}$$

(11) $$F1=\frac{2\times Precision\times Recall}{Precision+Recall}$$

(12) $$IoU=\frac{TP}{TP+FP+FN}$$

In Equations 8, 9, 10, 11 and 12, the TP , FP , FN , and TN denote the true positive (the area which is both predicted and annotated as root area), false positive (the area which is predicted as root area but annotated as background), false negative (the area which is predicted as background but annotated as root area) and true negative (the area which is both predicted and annotated as background) measurements. Accuracy is the proportion of the number of correctly predicted samples to the total number of samples. Recall is the proportion of samples that are correctly predicted as positive cases to all samples with true labels as positive cases. Precision is the proportion of samples correctly predicted as positive cases to all samples predicted as positive cases. F1 score is the harmonic mean of precision and recall. IoU is a commonly used measure in semantic segmentation to evaluate the overlap ratio of predicted results to ground truth.

## 3 Results and analysis

### 3.1 Training set loss and validation set loss

**Figure 7:**
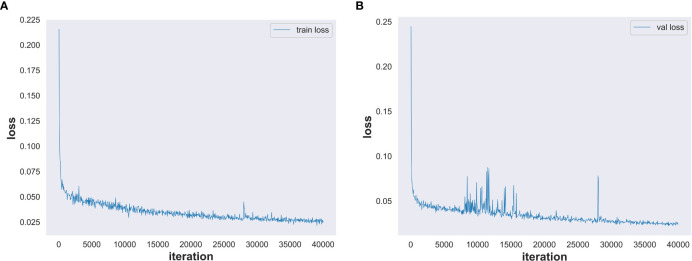
Training set loss and validation set loss. **(A)** Training set loss. **(B)** Validation set loss.

We set the number of model training iterations to 40,000 and used our high-resolution minirhizotron image dataset to train our improved OCRNet model. The total training time of the network model is 23.5 h. The training set loss is shown in **Figure 7A**. With the increasing number of training iterations, the training set loss decreased in the general trend. After 35,000 iterations, the training set loss was stabilized within 0.03, which indicates that the training set loss converged and the model was well trained. The validation set loss is shown in **Figure 7B**. With the increasing number of training iterations, the validation set loss also decreased under the general trend. Although the fluctuation of the validation set loss was a bit large in the middle two periods, it was within the reasonable range of the fluctuation of the validation set loss. After 35000 iterations, the validation set loss was stabilized within 0.029, which indicates that the validation set loss converged and stabilized.

### 3.2 Segmentation performance of improved OCRNet

**Figure 8:**
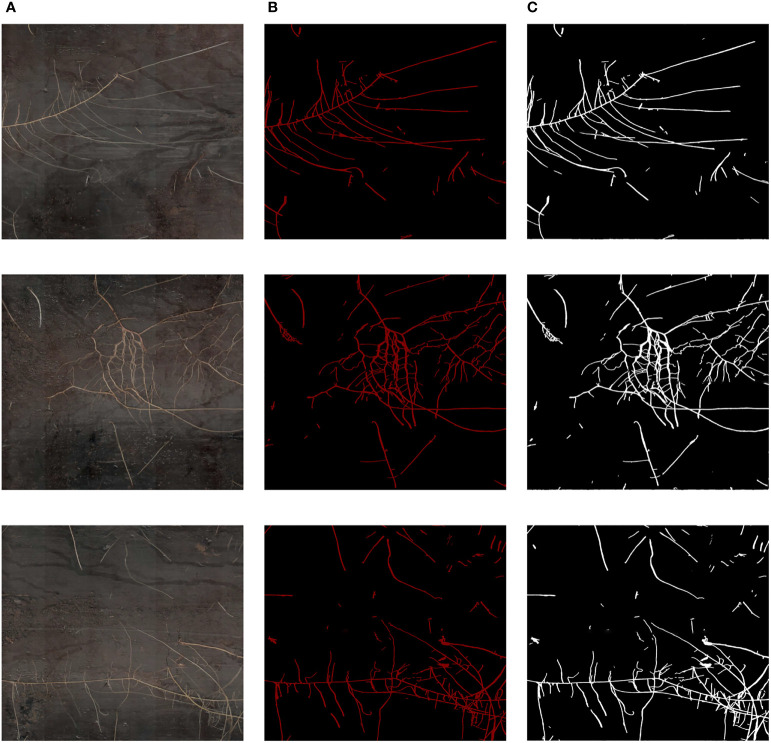
Comparison of our improved OCRNet segmented images and the corresponding annotated images. **(A)** Original images. **(B)** Annotated images. **(C)** Improved OCRNet segmented images.

In the process of training the network model, we saved the model weights every 500 iterations and tested the model weights using the validation set. Since IoU is one of the most common evaluation metrics in semantic segmentation, we selected the model weights with the highest IoU values for root segmentation in three randomly selected high-resolution minirhizotron images and compared the segmented images with the manually annotated images, as shown in **Figure 8**. Our improved OCRNet had good segmentation performance for root segmentation of the high-resolution minirhizotron images. In the segmentation results of these three randomly selected high-resolution minirhizotron images, many taproots and lateral roots were accurately identified and segmented. The overall segmentation results are very close to the annotated images.

### 3.3 Comparison with mainstream segmentation methods

To compare our improved method with the current mainstream semantic segmentation methods, we selected the original OCRNet, DeepLabv3+, PSPNet and FCN as comparison models, and the hyperparameters of each model during training were the same as those set during the training of our improved OCRNet. We used HRNetV2-W48 as the backbone of the original OCRNet and ResNet-50-C (He et al., 2019) as the backbone of DeepLabv3+, PSPNet and FCN. All comparison models were trained by using the high-resolution minirhizotron image dataset we produced. Similarly, we set the model weights to be saved and tested using the validation set every 500 iterations. The model weights with the highest IoU values for each semantic segmentation method when being tested using the validation set were used to evaluate the model segmentation performance by using the test set. As shown in **Table 2**, the five best model weights were used to obtain the values of each evaluation metric for each segmentation method, and the values of each evaluation metric were accurate to four decimal places. Although the precision values of our improved OCRNet model are slightly lower than that of DeepLabv3+, our improved OCRNet has the highest accuracy values, recall values, F1 score values and IoU values. The precision is the proportion of samples correctly predicted as positive cases to all samples predicted as positive cases. If there is a more serious under-segmentation of the model’s root segmentation results, then the precision may be high instead. Therefore, the precision cannot determine how well a model performs root segmentation. The IoU is a commonly used measure in semantic segmentation to evaluate the overlap ratio of predicted results to ground truth, so it is better than precision to evaluate the segmentation performance of a model. And our improved OCRNet achieved the highest IoU value of 0.8426. It illustrates that our improved semantic segmentation method has a stronger segmentation capability.

**Table 2:** Comparison between the different methods.

Method	Backbone	Accuracy	Recall	Precision	F1 score	IoU
FCN	ResNet-50-C	0.9826	0.9344	0.8511	0.8908	0.8031
PSPNet	ResNet-50-C	0.9826	0.9367	0.8495	0.8901	0.8034
DeepLabv3+	ResNet-50-C	0.9852	0.9121	**0.8949**	0.9034	0.8239
OCRNet	HRNetV2-W48	0.9859	0.9330	0.8875	0.9097	0.8343
Improved OCRNet	HRNetV2-W48	**0.9866**	**0.9419**	0.8887	**0.9146**	**0.8426**

Bold values indicates the maximum values in their columns.

**Figure 9:**
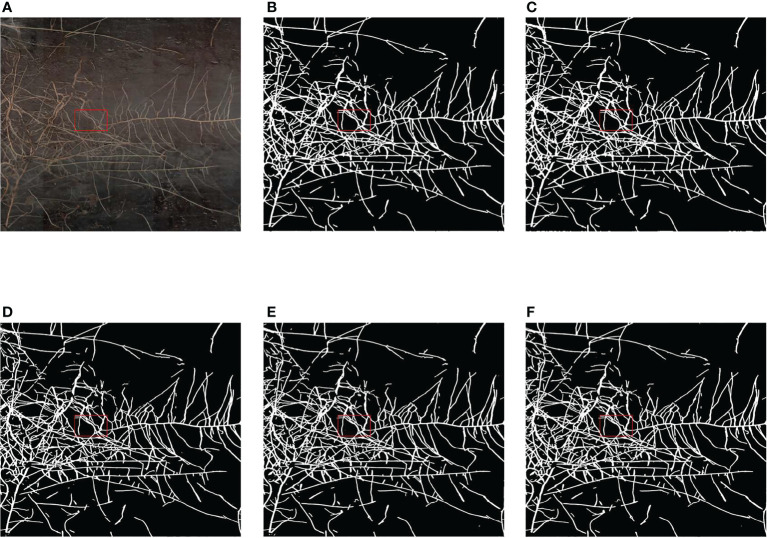
Comparison of the segmentation results of the five methods. **(A)** Original image. **(B)** Segmentation result of FCN. **(C)** Segmentation result of PSPNet. **(D)** Segmentation result of DeepLabv3+. **(E)** Segmentation result of OCRNet. **(F)** Segmentation result of improved OCRNet.

**Figure 10:**
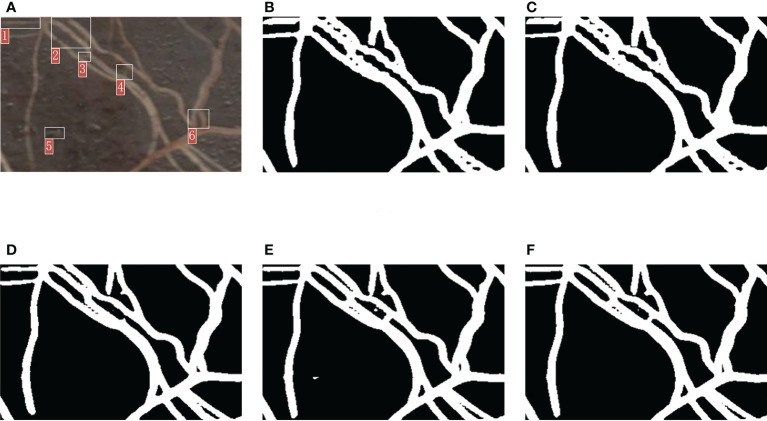
Comparison of the segmentation details of the five methods. **(A)** Original image. **(B)** Segmentation result of FCN. **(C)** Segmentation result of PSPNet. **(D)** Segmentation result of DeepLabv3+. **(E)** Segmentation result of OCRNet. **(F)** Segmentation result of improved OCRNet.

As shown in **Figure 9**, we selected a high-resolution minirhizotron image with very intricate roots to show the results of root segmentation of this image by using the five semantic segmentation methods in **Table 2**. As a whole, the integrity of the roots segmented in this image by each method is relatively high. Due to the high resolution of this root image, in **Figure 9**, we marked a red box in the original image as well as in the same location of each segmented image. Then, we zoomed in on the marked red box area of each segmented image to compare the segmentation details of each method, as shown in **Figure 10**.

In **Figure 10**, we marked six regions in the original image. At the position corresponding to region 1 in the original image, the root segmentation results of FCN and PSPNet were over-segmented, and the root contour segmented by DeepLabv3+ was very rough. At the position corresponding to region 2 in the original image, the root segmentation results of FCN and PSPNet were very rough because FCN and PSPNet identified some background parts of the two root gaps as root parts. At the position corresponding to region 3 in the original image, the root segmentation result of DeepLabv3+ was under-segmented. At the position corresponding to region 4 in the original image, the root segmentation results of FCN, PSPNet and DeepLabv3+ were under-segmented, and the root segmentation result of OCRNet was slightly over-segmented. At the position corresponding to region 5 in the original image, OCRNet identified the background noise as the root. At the position corresponding to region 6 in the original image, none of the five semantic segmentation methods segmented the gap between the roots very accurately, among which FCN and PSPNet did not identify the gap part between the roots basically. From the segmentation results of the six regions, there were more under-segmented regions in the root segmentation results for DeepLabv3+. This suggests that there may also be many under-segmented regions in the root segmentation results of DeepLabv3+ for other images, which may be the reason why the precision values of DeepLabv3+ are higher in **Table 2**. Overall, our improved OCRNet can pay attention to more root details and has better segmentation performance.

### 3.4 Segmentation performance in complex backgrounds

**Figure 11:**
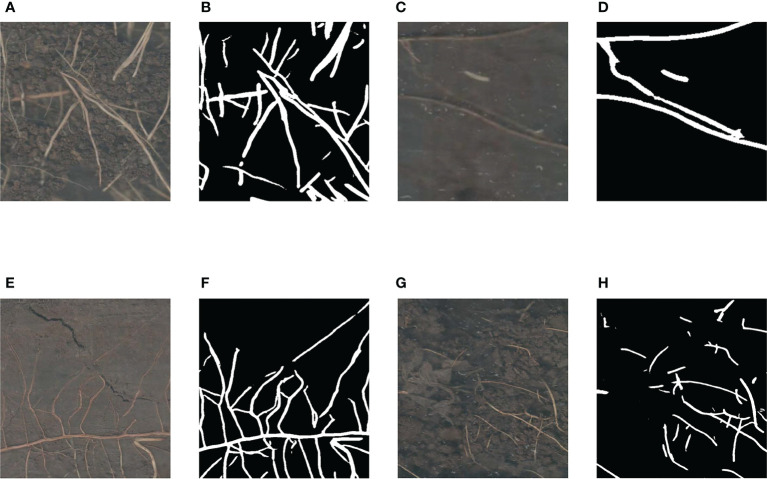
Various complex noises in the soil. **(A)** The region with stones in the background. **(B)** Segmentation result of the region with stones in the background. **(C)** The region with a white worm in the background. **(D)** Segmentation result of the region with a white worm in the background. **(E)** The region with some soil cracks in the background. **(F)** Segmentation result of the region with some soil cracks in the background. **(G)** The region with residual plastic film in the background. **(H)** Segmentation result of the region with residual plastic film in the background.

The root images we collected include a variety of complex background noises, such as stones, worms, soil cracks, residual plastic film, etc. We selected some regions of the root images with these background noises to show the results of our method to segment these regions, as shown in **Figure 11**. In **Figure 11A**, this region contains background noise such as stones, and in **Figure 11B**, our method segmented the roots of this region accurately and was largely unaffected by the stones in the background. In **Figure 11C**, this region contains a white worm, and in **Figure 11D**, although our method was also able to segment the roots in this region, it identified the body of this white worm as a root as well, which may be caused by the similarity of the body size and color of such white worms to the roots. In **Figure 11E**, there are some soil cracks in this region, and the shape of both the cracks and the roots are bar-shaped, and in **Figure 11F**, our method accurately separated the roots from the soil cracks. In **Figure 11G**, the left side of this region contains the residual plastic film in the soil, and in **Figure 11H**, our method classified only a very small portion of the residual plastic film as roots. Therefore, our method was able to distinguish the roots from other types of complex background noise relatively accurately, except for not identifying white worms as background noise. We believe that the small number of samples containing white worms in the background is also the reason why our trained model was unable to learn enough such cases to distinguish between roots and white worms correctly.

## 4 Discussion

The root images acquired in the real environments contain a variety of complex background noises. And these complex background noises can seriously interfere with automatic root identification and segmentation. Therefore, many root segmentation studies usually adopt the method of cultivating roots in ideal laboratory environments to exclude these interfering factors (Krzyzaniak et al., 2021; Xu et al., 2022; Zhao et al., 2022). The root images taken in ideal laboratory environments have less complex background noise, which is more favorable for root image segmentation. But the segmentation models trained by using the root images taken in ideal laboratory environments cannot meet the requirements for root segmentation in real soil environments. Minirhizotrons can be used to obtain *in-situ* root images non-destructively (Xu et al., 2020). To make our research applicable to actual production environments, we collected root images by minirhizotrons in a real soil environment.

The original images collected by minirhizotrons are basically high-resolution (Shen et al., 2020; Bauer et al., 2022). And there is usually not enough memory in the GPU to load high-resolution images for training due to the limited GPU memory (Gong et al., 2021). If the high-resolution images are resized so that the high-resolution images can be input into the network model for training, the resolution of the images will be reduced, and many details of the roots will be lost. It will prevent the model from learning enough root representations, resulting in a decrease in the model’s ability to distinguish the roots from the background. To solve this problem, we added a random cropping pipeline so that the root images were randomly cropped into sub-images of size 512 × 512 pixels before being input into the model for training. By adding a random cropping pipeline to reduce the size of the input images, the images were able to be input into the model for training while retaining the root details. However, in order to obtain high-level semantic information, in the process of extracting representations, the usual semantic segmentation model will first obtain low-resolution representations through down-sampling, and then the low-resolution representations will be restored to high-resolution representations by up-sampling, and this approach will lose a lot of valid information in the process of up-sampling and down-sampling (Sun et al., 2019b). Therefore, we used HRNetV2 as the backbone of the segmentation model to extract the root representations, which retained the high-resolution root representations through the parallel multi-resolution subnetworks in HRNetV2.

The traditional method of manually segmenting the roots in the images is very inefficient. Not only the speed of manual root segmentation is slow, but also the results of manual root segmentation may not be completely correct due to the visual fatigue problem in manual root segmentation (Abramoff et al., 2004; Le Bot et al., 2010). Therefore, an accurate and rapid root segmentation method is needed to replace inefficient manual root segmentation (Smith et al., 2020). In this study, our improved OCRNet model achieved automatic segmentation of roots in the soil and performed well in the root segmentation of the high-resolution minirhizotron images we acquired, achieving an accuracy of 0.9866, a recall of 0.9419, a precision of 0.8887, an F1 score of 0.9146 and an IoU of 0.8426, as shown in **Table 2**. And our improved OCRNet has the highest accuracy values, recall values, F1 score values and IoU values. It indicates the superiority of our improved method. It can be seen from **Figure 8** that the root segmentation results of our improved method are very close to the manually annotated images. Moreover, our method takes about 0.3 seconds to segment a root image of size 2271 × 2550 pixels, while we manually annotate the same root image in about 8 hours. It shows that our method is much faster than manual annotation. In terms of the accuracy of segmentation and the time taken for segmentation, our method has basically reached the level of replacing manual annotation.

**Figure 12:**
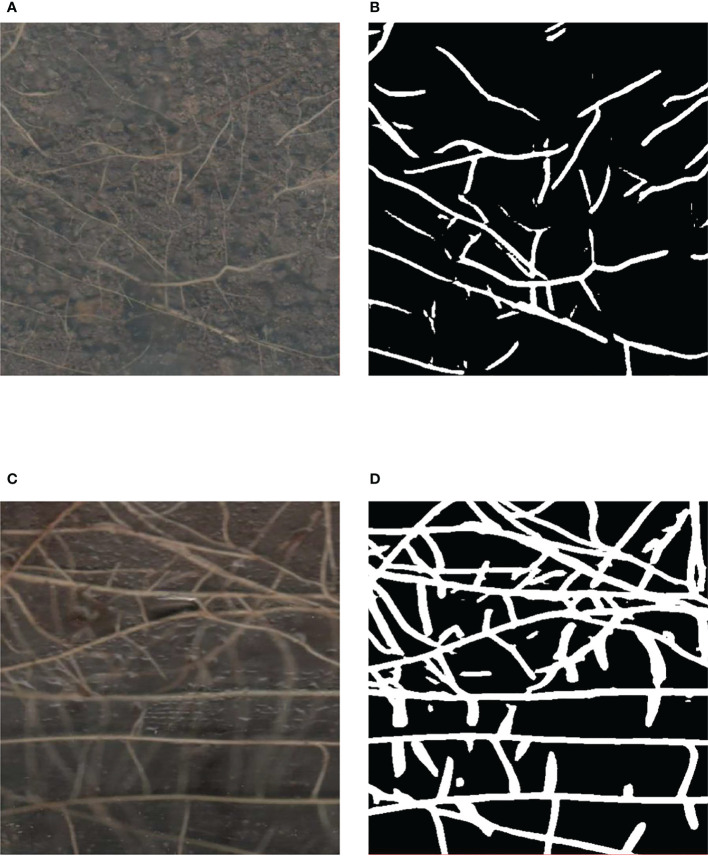
The filamentous root region and the light-colored root region. **(A)** The filamentous root region. **(B)** Segmentation result of the filamentous root region. **(C)** The light-colored root region. **(D)** Segmentation result of the light-colored root region.

Although our method has been able to achieve automatic root segmentation of high-resolution minirhizotron images taken under a real soil environment, it has some shortcomings in root segmentation. After comparing and analyzing the original images and the segmented images, we found that our method did not segment the filamentous roots and the light-colored roots accurately enough. As shown in **Figure 12**, we selected a region containing the filamentous roots and a region containing the light-colored roots to show the segmentation results of our method for these regions. In **Figure 12A**, this region contains many filamentous roots which are very fine and occupy very few pixels. In **Figure 12B**, the root parts in the segmentation result of our method for this part of the region were generally wider than those in the original image, and our method did not identify some very fine roots in the region. In our opinion, because of the small pixel area occupied by the filamentary roots, the model did not learn enough samples of these roots, which led to inaccurate segmentation results for these roots. In **Figure 12C**, this region contains many light-colored roots which are buried by the soil, so these roots are lightly colored in the image. In **Figure 12D**, our method did not identify some light-colored roots. It may be that the characteristics of the light-colored roots are not obvious, which makes it difficult for the model to accurately distinguish these roots from the background.

In the future, we will continue to collect and annotate more *in situ* cotton root images, especially those containing complex background noise, to expand our dataset. By expanding the dataset, the model will learn more samples, which will improve the generalizability of the model and the model’s ability to distinguish the roots from the background. Meanwhile, we will continue to improve our root segmentation model already improved in this paper to solve the problem of difficult segmentation of the filamentous roots and the light-colored roots by enhancing the superiority of the model framework.

## 5 Conclusion

To solve the problem of low efficiency of traditional manual root segmentation and to achieve automatic root segmentation of high-resolution minirhizotron images taken in a real soil environment, we improved the OCRNet by adding the GAM attention module and achieved accurate automatic segmentation of cotton roots using the improved OCRNet. Firstly, in order to make our research applicable to actual production environments, we collected images of roots in a real soil environment using minirhizotrons and produced a dataset. Then, we applied the OCRNet to the root segmentation of high-resolution minirhizotron images and selected HRNetV2 as the backbone of the OCRNet. Meanwhile, the structure of the original OCRNet was improved by adding the GAM attention module after the pixel representations output by HRNetV2, which made the pixel representations augmented. The pixel representations augmented by the attention mechanism were used to participate in the calculation of relevant parameters in the OCR module, which subsequently improved the ability of the model to distinguish the roots from the background. Next, we trained our improved OCRNet by using the high-resolution minirhizotron image dataset and set up a random cropping pipeline to preserve the details in the high-resolution minirhizotron images within the GPU memory limit. Finally, our improved OCRNet model achieved automatic segmentation of roots in the soil and performed well in the root segmentation of the high-resolution minirhizotron images we acquired, achieving an accuracy of 0.9866, a recall of 0.9419, a precision of 0.8887, an F1 score of 0.9146 and an IoU of 0.8426, as shown in **Table 2**. And our improved OCRNet has the highest accuracy values, recall values, F1 score values and IoU values. This method provided a new approach to automatic and accurate root segmentation of high-resolution minirhizotron images taken in the soil environments, which laid the foundation for automatic analysis of root phenotypic parameters in the field of root research.

## Data availability statement

The raw data supporting the conclusions of this article will be made available by the authors, without undue reservation.

## Author contributions

YH, JY, and YZ conceived the idea. PG, and XL contributed to the preparation of equipment and acquisition of data. YH wrote the code and tested the method. YH, and YZ validated the results. YH wrote the manuscript. JY, WY, CZ, PG, and XL revised the manuscript. All authors contributed to the article and approved the submitted version.
